# Annotations

**Published:** 1906-03-03

**Authors:** 


### ANNOTATIONS.

### A Plea for the Compulsory Notification of Pulmonary Tuberculosis.

There are few people, we imagine, who will cavil at the statement that pulmonary tuberculosis is a dangerous infective disease. By dint of constant pounding away the medical profession, with grateful assistance from the lay Press, which we are delighted to acknowledge, has achieved the general recognition of this fact. But much more remains to be done before one of the most preventible of diseases becomes a mere evil memory, and we welcome the narration in the columns of *The World* of an incident which will help forward the education of the public to the necessity for the notification of pulmonary tuberculosis. The incident related to the infection of a lady shortly after her removal to a flat. The physician under whose charge she came failed to find either in her family record or in her physical characteristics any sufficient explanation for her infection with tuberculosis, but discovered that the flat to which she had removed had, prior to her tenancy, been inhabited by a consumptive. The inference was that she had received the infection as a legacy from her predecessor, and, although the evidence is purely circumstantial, it cannot be denied that the inference is justified. In any case the flat, as our contemporary observes, "ought to have been thoroughly cleaned and disinfected, and the landlord ought to have seen to it before re-letting." The circumstances of the case afford a striking argument in favour of the notification of pulmonary tuberculosis, for such a proceeding would leave the landlord without option in the matter, and in this bad world too much freedom is often fatal to efficiency. Something has already been done in the direction indicated by the inauguration in several districts of a system of voluntary notification, but such a system does not reach far enough. No doubt the victims of tuberculosis of the lungs have much to put up with without being publicly branded as public dangers. But facts are often cruel, and "the greatest good of the greatest number" demands the small additional hardship involved in compulsory notification.

### Sleeping Out of Doors.

It seems probable that in the United States sleeping out of doors will soon become fashionable, and, no doubt, eventually, an attempt will be made to popularise the practice on this side of the Atlantic. Of course, the hygienic treatment for consumption has been in vogue for some years in America, but fresh developments have lately taken place, owing to the measures employed at the Presbyterian Hospital in New York with regard to pneumonia patients. It is stated that between 30 and 40 persons suffering from this disease, instead of being placed in a sheltered, well-warmed ward, have been located in the garden attached to the institution, carefully screened from the wind, but otherwise exposed to the air day and night; and that all the patients entirely recovered. The result achieved is the more notable because the average case mortality from pneumonia in New York is distinctly high. We are not surprised at the success which has resulted from this latest development of open-air treatment. The experience of numerous hospitals throughout the country is steadily diminishing the fears which have hitherto held us all in bondage, when sleeping out of doors is under consideration. It has been found that children and adults up to middle age, at any rate, may be allowed to sleep freely exposed to the air on hospital balconies without danger. As a matter of fact, they derive both benefit and pleasure from the nightly exposure, and can with difficulty bring themselves to return to their former habit of sleeping within doors. Seeing that both tuberculous patients, and those in average health, improve in condition when sleeping as well as spending the day in the open air, it requires no great effort of the imagination to suppose that others than healthy and tuberculous subjects may derive similar benefits from the same source. We believe that the time is not far distant when the benefits of open-air treatment will be extended as a matter of principle to many, if not the majority, of acute as well as chronic diseases, and, perhaps, to everyday life.

### Hygienic Shaving in Germany.

We lately commented on the dangers of being shaved even in what is known as a high-class hairdressing establishment, and advocated certain precautions which, in our opinion, should not be optional. The British barbers who did not appreciate our remarks may nevertheless do well to agree with the adversary quickly, and assent to the imposition of reasonable measures for the security of their customers lest a worse thing happen to them. In a large town in Germany the municipal authorities have put in force a series of regulations which, if unquestionably wholesome, may also be called wholesale. They embrace rules that the floor of the shop be washed twice a week; that head-rests be covered with a fresh piece of paper or linen for each customer; that bowls and shaving brushes be cleaned each time after being used; that soap be in the form of powder or small tablets; and that the barber wash his hands with warm water before attending to his customer, and be clad in long upper garments of light colour without pockets. Sponges, powder puffs, magnesia, and styptics in lumps, as well as revolving brushes, are entirely prohibited. It is also obligatory to clean scissors, brushes, and combs with ammonia, or soda, and an antiseptic solution. These rules are certainly admirably calculated to ensure hygienic shaving. If they were rendered compulsory in England, barbers would find their expenses so considerably augmented that they would almost be driven to the expedient of raising their prices. Most of us would, nevertheless, prefer a dear and clean shave to one that too often can only be faithfully described as cheap and nasty.

